# Absence of K13 Polymorphism in Plasmodium falciparum from Brazilian Areas Where the Parasite Is Endemic

**DOI:** 10.1128/AAC.00354-18

**Published:** 2018-09-24

**Authors:** Larissa Rodrigues Gomes, Aline Lavigne, Cassio Leonel Peterka, Patrícia Brasil, Didier Ménard, Cláudio Tadeu Daniel-Ribeiro, Maria de Fátima Ferreira-da-Cruz

**Affiliations:** a Laboratório de Pesquisa em Malária, Instituto Oswaldo Cruz, Fundação Oswaldo Cruz (Fiocruz), Rio de Janeiro, Brazil; b Centro de Pesquisa, Diagnóstico e Treinamento em Malária (CPD-Mal), Fiocruz, Rio de Janeiro, Brazil; c Programa Nacional de Prevenção e Controle da Malária, Secretaria de Vigilância em Saúde, Ministério da Saúde, Brasília, Brazil; d Laboratório de Doenças Febris Agudas, Instituto Nacional de Infectologia Evandro Chagas, Fiocruz, Rio de Janeiro, Brazil; e Malaria Genetic and Resistance Group, Biology of Host-Parasite Interactions Unit, Institut Pasteur, Paris, France

**Keywords:** artemisinin, kelch domain, malaria, P. falciparum, Brazil, K13, chemoresistance

## Abstract

Plasmodium falciparum artemisinin-resistant parasites can be evaluated by examining polymorphisms in the kelch (*PfK13*) domain. A total of 69 samples from patients with falciparum malaria were analyzed. All samples were from areas in states in Brazil where the parasite was endemic: Acre (*n* = 14), Amapá (*n* = 15), Amazonas (*n* = 30), and Pará (*n* = 10). After DNA alignment with the 3D7 reference sequence, all samples were found to be wild type. These data provide a baseline for *PfK13* and reinforce the pertinence of artemisinin combination therapy in Brazilian areas.

## Abstract (precis)

Plasmodium falciparum artemisinin-resistant parasites can be evaluated by examining polymorphisms in the kelch (*PfK13*) domain. A total of 69 samples from patients with falciparum malaria were analyzed.

## TEXT

*P*lasmodium falciparum resistance to different antimalarial drugs is a serious obstacle to malaria elimination. The first clinical cases of artemisinin (ART) resistance were reported in western Cambodia (1), followed by the detection of P. falciparum parasites with reduced *in vivo* susceptibility to artesunate (2, 3). Since then, resistance to ART derivatives has emerged or spread throughout Southeast Asia (4–9). In addition, a delayed clearance of P. falciparum parasites was noted recently in Equatorial Guinea (10).

In South America, C580Y mutant parasites emerged in Guyana, independently from those detected in Southeast Asia (11). In fact, ART combined therapy (ACT) systematic self-medication by gold miners in French Guiana together with illegal miner movement through the Brazil-French Guiana border represent a serious risk for the emergence of ART resistance in Guiana Shield and its consequent spread to the Brazilian area where the parasites are endemic (12).

Brazil has achieved the goal of reducing the number of falciparum malaria cases and now intends to enter the pre-elimination stage (13). In such a low-transmission area prone to emergence of resistance, it is urgent to monitor ACT efficacy and identify early warning signs of ART-resistant P. falciparum parasites. Genome association studies strongly linked a *locus* on P. falciparum chromosome 13 to ART resistance in the kelch propeller domain (14). To date, almost 200 K13 mutations have been described worldwide, but only 6 nonsynonymous single nucleotide polymorphisms were associated with ART resistance (15). The purpose of this work was to assess polymorphisms in the *Pfk13* gene in Brazilian isolates from patients who attended the National Reference Centre for Diagnostics and Training in the extra-Amazon region (Centro de Pesquisa, Diagnóstico e Treinamento em Malária [CPD-Mal]), Fundação Oswaldo Cruz (Fiocruz), Rio de Janeiro, Brazil.

Blood samples were collected between 2010 and 2017 from febrile patients who visited areas where the parasite was endemic and who attended the clinic for diagnosis and treatment for malaria at the Ambulatório de Doenças Febris Agudas, INI-IPEC, Fiocruz, in Rio de Janeiro where malaria transmission does not occur. Therefore, all the individuals presented with malaria that was caught in Brazilian Amazon localities where the parasite is endemic. The study was performed at the Laboratório de Pesquisa em Malária, headquarters of the CPD-Mal of Fiocruz. Inclusion criterion comprised patients with a microscopic and/or molecular diagnosis of falciparum malaria. After obtaining patient informed consent, venous blood was collected according to protocols previously approved by the Ethical Research Committees of Fiocruz (32839013.6.00005248) before starting malarial therapy. P. falciparum samples were diagnosed by Giemsa-stained thick and thin smears and by PCR (16). Genomic DNA was isolated from 1 ml whole blood using QIAamp midi columns (Qiagen), as described by the manufacturer. All patients were treated with 3 days of a fixed artesunate-mefloquine combination, according to Brazilian National Malaria Program guidelines (17), and followed at least until parasite clearance and, whenever possible, up to 42 days. The *PfK13* gene fragment was amplified by nested PCR using previously published primers (14). Cambodian isolates with known K13 mutations (provided by D. Ménard) were employed in each PCR run as quality/positive controls. DNA sequencing was carried out after purification using the Wizard SV gel and PCR clean-up system (Promega). Briefly, the amplified fragments were sequenced using BigDye Terminator cycle sequencing ready reaction version 3.1 and ABI Prism DNA analyzer 3730 (Applied Biosystems) (18) at the Genomic Platform/PDTIS/Fiocruz. Polymorphisms in the *PfK13* gene were analyzed by direct DNA sequencing of amplicons, and the obtained sequences (from codon 443 to 666, i.e., 720 bp) were aligned with the *PfK13*-propeller region (GenBank accession no. NC_004331.3) of the 3D7 reference sequence (PF3D7_1343700), using the free software BioEdit sequence alignment editor version 7.2.5.

A total of 69 patients (aged 19 to 55 years) were diagnosed with falciparum malaria by microscopic and molecular tests. The parasitemia ranged from 2,340 to 288,000 parasites/μl (*x* = 88,040). All patients with malaria presented with clinical signs or symptoms of uncomplicated malaria, such as fever, headache, and chills, and the baseline characteristics were similar. Patients from Acre showed higher parasitemias (Table 1).

**TABLE 1:** Number of cases, parasitemia, and sex of 69 uncomplicated falciparum malaria patients, by to Brazilian states

Parameter	State			
	Amazonas	Amapá	Acre	Pará
No. of cases	30	15	14	10
No. of parasites/μl (mean)	11,818	7,715	48,794	15,790
Male/female	17:13	5:10	8:6	4:6

Although the study was not designed to determine clinical drug efficacy, all treated patients were followed clinically and through parasitological and molecular examinations. On the third day of ACT, all patients had negative parasitological and molecular results, and no recrudescence was recorded after the treatment period; reinfections did not occur because the patients were in Rio de Janeiro, out of the areas where the parasite was endemic. All samples were from Brazilian areas in states where the parasite was endemic, i.e., Acre (*n* = 14), Amapá (*n* = 15), Amazonas (*n* = 30), and Pará (*n* = 10). P. falciparum DNA was successfully sequenced in all 69 isolates. After alignment with the 3D7 reference sequence, all samples were found to be wild type.

Novel K13 nonsynonymous polymorphisms were observed in Africa (19), including 9 sub-Saharan countries (20), Equatorial Guinea (10), and Mozambique (21), highlighting the need for continuous monitoring of drug resistance in Africa.

In the Brazil/French-Guiana border region, the existence of mobile populations engaged in gold mining, logging, or illegal activities with risk of malarial transmission raises the potentiality of spreading ART resistance alleles with serious implications for P. falciparum surveillance, malaria control, and elimination efforts in Brazil. Indeed, a study conducted in illegal gold miners in French Guiana showed that most of the recruited participants were from Brazil (93.8%), and PCR-based methods revealed P. falciparum isolates in 47.9% of the cases (22). In this work, all P. falciparum isolates collected before ACT (day 0) exhibited the 3D7 wild-type allele in the propeller region of the *PfK13* gene, including those from the Brazil-Guiana Shield border (Amapá). In an analogous study, with samples only from Acre, no polymorphism was found in patients with parasitological and clinical cure (23). Clinical ART resistance is defined as a reduced parasitic clearance rate, expressed as an increased parasite clearance half-life or persistence of microscopically detectable parasites on the third day of ACT (2, 5). Here, no parasite was observed on day 3, and no treatment failure was detected. The lack of mutations in the *K13* gene of P. falciparum parasites from Brazilian areas where the parasite is endemic is in agreement with the adequate clinical and parasitological responses, supported by PCR results, showing the efficacy of ACT. Although an *in vitro* ring-stage survival assay (RSA) was not performed, it is well known that the parasite clearance half-life parameter correlates strongly with RSA results. Consequently, the present data contribute to the ongoing surveillance of ART resistance parasites by providing baseline data on K13-propeller mutations and reinforce the pertinence of the use of ACTs in Brazilian areas where the parasite is endemic.

Similar studies with a larger number of samples will help ascertain any emergence of ART resistance, and routine monitoring must continue to ensure that the ACTs are effective in the treatment of falciparum malaria in Brazilian areas where the parasite is endemic.
